# New report of *Eratyrus cuspidatus* Stål, 1859 (Hemiptera: Reduviidae: Triatominae) in the State of Campeche, Mexico

**DOI:** 10.1590/0037-8682-0299-2019

**Published:** 2020-01-27

**Authors:** Paulino Tamay-Segovia, Selene Blum-Domínguez, Ricardo Alejandre-Aguilar, Luis Alberto Núñez-Oreza, Betty Sarabia-Alcocer, Vicente Jesús Chan-Puc

**Affiliations:** 1 Laboratorio de Enfermedades Transmitidas por Vectores y Zoonosis. Centro de Investigaciones Biomédicas de la Universidad Autónoma de Campeche. Campeche, México.; 2 Laboratorio de Enfermedades Tropicales, Centro de Investigaciones Biomédicas de la Universidad Autónoma de Campeche. Campeche, México.; 3 Laboratorio de Entomología, Depto. de Parasitología Escuela Nacional de Ciencias Biológicas, Instituto Politécnico Nacional. Ciudad de México, México.; 4 Laboratorio de Microbiología y Biología Molecular, Centro de Investigaciones Biomédicas de la Universidad Autónoma de Campeche. Campeche, México.; 5 Facultad de Medicina de la Universidad Autónoma de Campeche. Campeche, México.; 6 Facultad de Enfermería de la Universidad Autónoma de Campeche. Campeche, México.

**Keywords:** Triatominae, Eratyrus cuspidatus, Sylvatic vectors

## Abstract

**INTRODUCTION:**

Triatomine bugs are vectors of *Trypanosoma cruzi*, the etiological agent of Chagas disease.

**METHODS:**

Triatomine bugs were collected and identified following established protocols. In addition, infection with *T. cruzi* was detected by microscopic and molecular analysis.

**RESULTS:**

We captured an adult male specimen of the *Eratyrus cuspidatus* species that has not been reported in the state of Campeche.

**CONCLUSIONS:**

This finding provides new information on the distribution of *E. cuspidatus* in Mexico. However, more studies are needed to determine their epidemiological significance.

Triatomine bugs are important vectors of *Trypanosoma cruzi* (Chagas, 1909), the etiological agent of Chagas disease, which is endemic across much of the Americas. According to the Pan American Health Organization, approximately 6 million people are infected and 65 million are at risk of contracting the infection^1^. Currently, 150 extant and 2 extinct species of triatomines are known, and grouped into 16 genera^2^. In Mexico, approximately 33 triatomine (Hemiptera: Reduviidae: Triatominae) species have been reported, so far. It is therefore, one of the countries with the greatest diversity of triatomines. Two genera namely, *Triatoma* (20 species) and *Meccus* (6 species) are the most abundant and widely distributed^3^. However, other species, that until recently, had not been considered epidemiologically significant have also been reported^3^: *Dipetalogaster maxima* (Uhler, 1894), *Eratyrus cuspidatus* (Stål, 1859), *Paratriatoma hirsuta* (Barber, 1938), *Panstrongylus rufotuberculatus* (Champion, 1899), *Triatoma nitida* (Usinger, 1939), and *Rhodnius prolixu*s (Stål, 1859). *E. cuspidatus* was found to be naturally infected with *T. cruzi* ^4^ ^,^ ^5^ ^,^ ^6^. They have begun to invade and colonize human dwellings. Since they transmit the etiologic agent of Chagas disease, more knowledge is needed about their distribution and ecology, to improve control strategies.

*E. cuspidatus* is distributed throughout Colombia, Ecuador, Guatemala, Mexico, Panama, Peru, and Venezuela^3^. In Mexico, it has been observed only in the three states: Chiapas, Veracruz, and Yucatan^3^ ^,^ ^7^ ^,^ ^8^ ^,^ ^9^. In the state of Campeche, *Triatoma dimidiata* (Latreille, 1811) is the only species associated with *T. cruzi* transmission, since the last 70 years^4^. However other vector species have been identified in neighboring states. It is therefore, likely that more than one vector is present in Campeche^3^ ^,^ ^7^.

Reports of sylvatic species of triatomines, contribute to the knowledge about current and new species distributions, and describe their role as existing or possible vectors of *T. cruzi*. 

The state of Campeche is located within the following geographic coordinates: 17°48’46” N to 20°50’53” N and 92°28’7” W to 89°07’16” W. It is bordered to the north by the Gulf of Mexico and Yucatan, to the east by Quintana Roo and Belize, to the south by the Republic of Guatemala and the state of Tabasco, and to the west by the state of Tabasco and the Gulf of Mexico. The average annual temperature throughout the state is 26 °C while that along the coast is 28 °C^10^.

In March 2010, a study was conducted in Calakmul, a protected, natural, rainforest reserve, located in the Calakmul municipality, approximately 179.5 km from the capital city (Campeche). Triatomine bugs were collected manually, during the day, and with a lamp at night, according to the method described by Schofield^11^. During the search, we examined all possible ecotopes (including hollow trees and cracks, holes in the ground, and stone piles). Captured specimens were placed in labeled flasks, containing folded cardboard sheets, and transported to the laboratory for morphological identification according to the key described by Lent and Wygodzinsky^4^. Infection with flagellates was determined by microscopic observation of feces, obtained after abdominal compression, and dilution in phosphate buffered saline (PBS). Presence of *T. cruzi* was determined by PCR^12^. 

Primarily, adults of *T. dimidiata* infected with *T. cruzi* were collected. Although this species was the most abundant, one adult of another triatominae species was collected. This specimen was probably attracted to the lights and consequently, captured near the bedroom, of the home of forest guards (18°21'54'' N and 089°53'32'' W) and visitors. It was identified as a male of *E. cuspidatus* ^4^, which presents rounded discal tubercles, humeral angles angular or pointed but not spinose, and the process of the scutellum bent slightly upward (Figure 1). In contrast *E. mucronatus* presents discal tubercles in the form of strong spines, humeral angles distinctly spinose, and the scutellum upward in variable angle. The authors also compared the specimen with *T. dimidiata,* the more abundant vector in the area. *T. dimidiata* presents discal tubercles that are not pointed and not elevated, humeral angles rounded, a scutellum with a central area that is not depressed, and an apical process that is subcylindrical and bent slightly downward at the apex (Figure 2B). Identification of *E. cuspidatus* was confirmed by the entomologist, Alejandre-Aguilar, expert in triatomines, at the Laboratory of Entomology of the Escuela Nacional de Ciencias Biologicas del Instituto Politecnico Nacional. 

**FIGURE 1:**
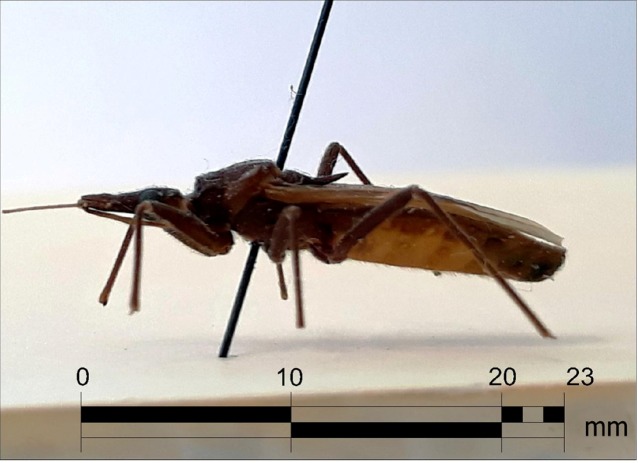
Lateral view of a male specimen of *Eratyrus cuspidatus*, length 23mm.

**FIGURE 2:**
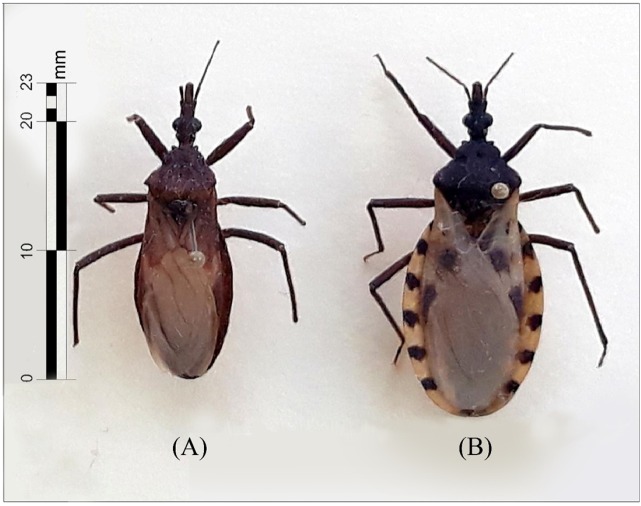
Dorsal view of two male triatomine specimens: **(A)** *Eratyrus cuspidatus*, length 23mm. and **(B)** *Triatoma dimidiata*, 26mm.

The most important epidemiological species are those that live among humans and have a strong preference for human blood^11^. However, sylvatic species are a source of infestation and re-infestation; in some cases, they even replace domestic species that had been controlled with chemical products (insecticides). When they adapt to the type of habitat and fauna, they can establish an ecological chain of epidemiologically important vector species^13^. Thus, *E. cuspidatus* could contribute to the transmission of *T. cruzi* in humans, as reported in other countries^14^.

In addition to *T. dimidiata* reported by Valdez-Tah et al.^15^, the occurrence of *E. cuspidatus* in proximity to a human dwelling suggests, that it may eventually become a part of the transmission cycle of *T. cruzi* among the human communities established in these areas. 

This study reports the occurrence of a particular species that had not been previously reported in the state. It also reports the recent proximity of this species to human environments, possibly as a consequence of human movement into sylvatic areas.

We examined *E. cuspidatus* for *T. cruzi* infection. However, no flagellates were detected. Similarly, the result of PCR was also negative for the presence of *T. cruzi*. Thus, our findings concur with other reports of *E. cuspidatus* in Mexico. However, it is yet unknown, whether they are limited to sylvan areas, with occasional intrusion into human dwellings, or if they could possibly invade domestic areas. Therefore, more studies are needed to further investigate and determine the importance of these species as vectors of *T. cruzi*. 
